# Transactivation of TrkB Receptors by Oxytocin and Its G Protein-Coupled Receptor

**DOI:** 10.3389/fnmol.2022.891537

**Published:** 2022-06-02

**Authors:** Mariela Mitre, Khalil Saadipour, Kevin Williams, Latika Khatri, Robert C. Froemke, Moses V. Chao

**Affiliations:** ^1^Departments of Cell Biology, Neuroscience & Physiology, and Psychiatry, Skirball Institute for Biomolecular Medicine, New York, NY, United States; ^2^Neuroscience Institute, New York University Langone Medical Center, New York, NY, United States; ^3^Departments of Cell Biology, Psychiatry, New York University Langone Medical Center, New York, NY, United States; ^4^Department of Neuroscience and Physiology, New York University Langone Medical Center, New York, NY, United States; ^5^Department of Otolaryngology, New York University Langone Medical Center, New York, NY, United States; ^6^Center for Neural Science, New York University, New York, NY, United States; ^7^Departments of Biology and Psychology, University of Georgia, Athens, GA, United States

**Keywords:** oxytocin, transactivation, receptor tyrosine kinase receptors, neurotrophin, trophic

## Abstract

Brain-derived Neurotrophic Factor (BDNF) binds to the TrkB tyrosine kinase receptor, which dictates the sensitivity of neurons to BDNF. A unique feature of TrkB is the ability to be activated by small molecules in a process called transactivation. Here we report that the brain neuropeptide oxytocin increases BDNF TrkB activity in primary cortical neurons and in the mammalian neocortex during postnatal development. Oxytocin produces its effects through a G protein-coupled receptor (GPCR), however, the receptor signaling events that account for its actions have not been fully defined. We find oxytocin rapidly transactivates TrkB receptors in bath application of acute brain slices of 2-week-old mice and in primary cortical culture by increasing TrkB receptor tyrosine phosphorylation. The effects of oxytocin signaling could be distinguished from the related vasopressin receptor. The transactivation of TrkB receptors by oxytocin enhances the clustering of gephyrin, a scaffold protein responsible to coordinate inhibitory responses. Because oxytocin displays pro-social functions in maternal care, cognition, and social attachment, it is currently a focus of therapeutic strategies in autism spectrum disorders. Interestingly, oxytocin and BDNF are both implicated in the pathophysiology of depression, schizophrenia, anxiety, and cognition. These results imply that oxytocin may rely upon crosstalk with BDNF signaling to facilitate its actions through receptor transactivation.

## Introduction

Oxytocin is an evolutionarily conserved neuropeptide with profound effects on maternal care and social behavior. Synthesized from the paraventricular and supraoptic nucleus of the hypothalamus, it is released both peripherally and centrally. Much attention has been given to how oxytocin transduces signals peripherally during parturition and lactation in the myometrium, as well as centrally (Gimpl and Fahrenholz, 2001; Arrowsmith and Wray, 2014). Both G_i_ and G_q_ protein-mediated coupling is associated with stimulatory and inhibitory effects of oxytocin (Busnelli et al., 2012). Of note, oxytocin also has significant effects in the brain that mediate various forms of social cognition and social reward (Insel and Young, 2001; Bartz et al., 2011; Dolen et al., 2013; Marlin et al., 2015) in addition to anxiolytic effects upon stress and anxiety (Yoshida et al., 2009). Oxytocin is typically known due to its effect on social behavior, specifically bonding. The interest originated after observations of mother-infant bonding, which are changed by levels of oxytocin (Zik and Roberts, 2015; Shamay-Tsoory and Young, 2016). Other reactions, such as motivation, recognition of others, and trust are affected as well. How oxytocin conveys downstream signals to account for its specific physiological and behavioral effects is not fully understood.

Recent studies found that oxytocin influences the expression of neurotrophic factors, such as Nerve Growth Factor (NGF) and BDNF, which can contribute to trophic effects and neural plasticity (Park and Poo, 2013; Bakos et al., 2018). For instance, in the presence of oxytocin, BDNF mRNA expression is highly regulated in primary hippocampal neurons (Zhang et al., 2020).

Emerging evidence shows that oxytocin and BDNF regulate a range of physiological processes, including maternal behavior (Maynard et al., 2018), cell metabolism, eating behavior, and obesity (Kernie et al., 2000; Marosi and Mattson, 2014). As an activity-dependent trophic factor, BDNF has a widespread role as a mediator of adaptive responses of the brain and body to fluctuations in energy intake and expenditure, and homeostasis. Likewise, oxytocin is an immediate and direct modulator of energy expenditure and glucose metabolism in peripheral and central sites (Ott et al., 2013; Lawson, 2017).

To mediate its diverse effects, oxytocin acts through a single G protein-coupled receptor encoded by a single gene. In the brain, the receptor for oxytocin is expressed at time periods of heightened plasticity, which is also promoted by BDNF, an activity-dependent trophic factor. Oxytocin receptors are highly expressed in the cortex at 2 weeks of mouse postnatal life, similar to the expression pattern of BDNF. Receptors for oxytocin and BDNF overlap in similar brain regions, especially in areas important for learning and memory. An important aspect of oxytocin’s effects on the brain is its expression during periods of plasticity, which also occur with BDNF signaling during postnatal periods (Maynard et al., 2018). Many brain regions such as the hippocampus and cortex exhibit activity-dependent enhancement of BDNF, that are lowered by oxytocin receptor antagonists (Zhang et al., 2020). These observations suggest that interconnections exist between oxytocin and BDNF.

The BDNF TrkB receptor is found throughout the nervous system during development and in adult stages, including the cortex, thalamus, hippocampus, amygdala, lateral septal nucleus, and cerebellar cortex (Parada et al., 1992; Masana et al., 1993). The distribution of the oxytocin receptors in the mouse central nervous system (CNS) using an antibody specific for the oxytocin receptor likewise showed strong expression postnatally in the hippocampus, lateral septum, nucleus accumbens, amygdale, and many regions of the cortex (Mitre et al., 2016). The expression of receptors for oxytocin and BDNF, therefore, has overlaps in many brain regions, especially in areas important for learning and memory. Peak expression in the cortex occurs during the 2nd and 3rd postnatal weeks, a time during which trophic factors such as BDNF are critical for the maturation of inhibitory circuits (Rico et al., 2002).

Both BDNF and oxytocin exert an influence on psychiatric disorders, such as social behavior, autism, schizophrenia, and mood and anxiety disorders (Autry and Monteggia, 2012; Yoon and Kim, 2020). As oxytocin and BDNF receptors are abundantly found in the cortex and hippocampus (Owen et al., 2013; Mitre et al., 2016), these observations suggest that oxytocin may have an effect on BDNF trophic factor signaling during postnatal cortical development. We have therefore investigated the possibility that receptors for oxytocin and BDNF may be connected to their signaling pathways.

## Materials and Methods

### Animals and Primary Neuron Cultures

All experiments are conducted in accordance with the guidelines of the NIH (DHHS Guide for the Care and Use of Laboratory Animals: 1985) and the Guidelines for the Use of Animals in Neuroscience Research by the Society for Neuroscience. The animal research experiments were approved according to the NYU Institutional Animal Care and Use Committee (IACUC) of New York University Langone Medical Center. Primary cortical neurons were isolated from E18 mice, cultured on poly-D-Lysine coated coverslips or 6-well plates, and maintained for a week *in vitro* in a neurobasal medium containing B27 supplement, 0.5 mM L-glutamine.

### Brain Slices

Brain slices were collected from the somatosensory cortical area, piriform cortex, and auditory and incubated in oxygenated artificial cerebrospinal fluid and collected at 15 min, 30 min, or 1 h after oxytocin treatment or incubation alone in the control chamber. Lysates were prepared and run *via* Western blotting to probe for pTrkB (Y816) and total TrkB.

### Cell Culture and Transfection

Human embryonic kidney 293 (HEK-293-TrkB) cells were obtained from Hiroyuki Nawa (Narisawa-Saito et al., 2002). The cells were maintained in DMEM containing 10% fetal bovine serum supplemented with 100 U/ml penicillin, 100 mg/ml streptomycin, 2 mM glutamine, and 200 mg/ml G418. The cells were transfected with different concentrations of plasmids (pcDNA OXTR-myc, pcDNA OXTR-Venus, or pcDNA V2R-myc) for 24 h using Lipofectamine 2000. Transfected cells were fixed with 4% paraformaldehyde, permeabilized with 0.2% Triton X, blocked and stained with primary antibody followed by secondary antibody.

### Antibodies

The pTrkB polyclonal antibody recognized tyrosine-816 at the C-terminus of TrkB. This antibody is specific to pTrkB and did not cross-react with the non-phosphorylated receptor protein. The phosphorylation of TrkB by oxytocin occurs within 20–30 min to 2 h. The measurements of the dose-response relationship suggested that oxytocin treatments of 0.1 μM and 1 μM were the most effective at transactivating TrkB receptors and were used further in the biochemical analysis. Antibodies used in this study are listed below:

**Table:** 

phospho-TrkB (Y816)	Made in the Chao lab (Rajagopal et al., 2004; Arevalo et al., 2006)
*p*-TrkB	Cell Signaling (#4618)
RatTrkB	mouse monoclonal antibody BD Transduction (#610102)
Total TrkB	Goatantibody R&D Systems (#AF1494)	
p-MAPK/ERK	Cell Signaling (#9102)
c-myc 9E10	epitope antibody Cell Signaling(#2276S);
VGAT	Synaptic Systems (#131011)
gephyrin	monoclonal mouse antibody (Abcam #Ab25784).
MAP2	Chicken antibody Covance (PCK-554#x02013;050)	
OXTR-2	Rabbit polyclonal antibody (Mitre et al., 2016), see below

The OXTR-2 antibody was generated against the following sequences EGS-DAAGGAGRAALARVSSVKLISKAKI (aa 243–270; OXTR-2) in the third intracellular loop. These target peptides were chosen based on a high level of antigenicity using the Thermo Scientific Antigen Profiler. We identified epitopes that were maximally different from corresponding regions of the vasopressin V1a/V1b receptors. BLAST analysis was performed to ensure the uniqueness of these sites in the mouse proteome. The peptides were generated by AnaSpec with a terminal cysteine added to each peptide to allow for conjugation to key-hole limpet hemocyanin (KLH). KLH conjugation and injection into rabbits were performed by the Pocono Rabbit Farm and Laboratory. Each rabbit was injected with the respective peptide solution biweekly and a small bleed was collected monthly. The resulting polyclonal antisera were tested using dot and Western blot and immunohistochemistry before being further purified using affinity chromatography. Specificity of antibodies was evaluated *via* Western blot analysis of HEK293 cells expressing an oxytocin receptor–Venus construct (provided by L.J. Young, Emory University) and protein extracts from mouse uterus and brain. Untransfected HEK293 cells, as well as tissues from the oxytocin-receptor knock-out uterus and brain, were used as controls in each Western blot analysis. e compared the immune and preimmune sera by immunoblotting with different amounts of pure target OXTR-2 peptide. No reactivity was observed in the preimmune bleeds, but higher levels of reactivity were observed with increasing amounts of OXTR-2. We then used HEK293T cells transfected with OXTR-IRES-Venus cDNA to express oxytocin receptors and the Venus YFP variant in the same cells. In Western blots from oxytocin-receptor-expressing HEK cells, we observed a protein band at 43 kDa that corresponded to the expected molecular weight of the mouse oxytocin receptor.

### Immunostaining

Both brain sections and cortical neurons fixed on coverslip were incubated with primary antibodies in donkey serum/PBS/Triton at 4°C overnight, followed by incubation with secondary fluorescence antibodies. pTrkB immunoreactivity in primary cortical neurons was revealed using Alexa-Fluor 488 as the immunolabel. DAPI was used as a fluorescent stain for DNA (Molecular Probes, Hoechst).

### RNA Isolation and Reverse-Transcription PCR (RT-qPCR)

Total RNA was extracted from the cortex, hypothalamus, and HEK-293TrkB cells using Trizol. cDNA was generated using qScript cDNA SuperMix (QuantaBio). Quantitative real-time PCR (qRT-PCR) analysis was performed using SYBR Green PCR Master Mix (Applied Biosystem) and a StepOnePlus Real-Time PCR System (Applied Biosystem). Primers from Yang et al. (2017) are listed below.

OXT_hum_for. 5’-GCTGAAACTTGATGGCTCCG-3’

OXT_hum_rev. 5’-TTCTGGGGTGGCTATGGG-3’

Actin_hum_for. 5’-TCCCTGGAGAAGAGCTACGA-3’

Actin_hum_rev. 5’-TGAAGGTAGTTTCGTGGATGC-3’

Actinb_ms_for

5’-TGTGACGTTGACATCCGTAAA-3’

Actinb_ms_rev

5’ GTACTTGCGCTCAGGAGGAG-3’

OXT_ms_Fw

5’-GAGGAGAACTACCTGCCTTCG-3’

OXT_ms_Rv

5’-TCCCAGAAAGTGGGCTCAG-3’

### Rigor and Reproducibility

Mouse samples for cortical slices and primary neuronal cultures are: (i) matched for experimental and control groups for age and sex; (ii) randomized the selection of mice; (iii) and excluded mice with abnormalities; additionally; and (iv) the cell line experiments were reproduced at least three times. We are aware of the potential variability in the results due to sex and age differences. We have included a similar number of males and females of the same age in our study design. For example, in previous proteomic studies, we will examine dams and male C57BL/6 mice (*N* = 12, six of each sex). There are many mechanisms that may account for TrkB receptor activation through oxytocin. We are aware that transactivation may not be responsible for all the actions of oxytocin. One possibility is the trophic effects of oxytocin, which might be mediated by TrkB receptor signaling.

### Statistical Analysis

The results represent the average of at least three independent experiments unless indicated. Statistical significance was determined by One-way repeated measures of variance (ANOVA) or Student’s *t*-test. All statistical analyses of quantitative data are expressed as mean ± SEM. The expression of oxytocin receptors was measured by the use of receptor antibodies generated in our lab. Trk receptors were measured by anti-phosphotyrosine antibodies. Quantification of immunolabeled sections is performed in a blinded fashion on images with statistical analysis. For each protein measurement, the statistical significance is measured. The data for immunofluorescence and western blot to used Graph pad (Prism) or Image J to calculate statistics. Depending on the results, we utilized ANOVA tests followed by *post-hoc* Tukey. This analysis was specifically described in each Figure legend.

The *p* values equal to or less than 0.05 were considered significant, asterisks denote statistical significance (*p < 0.05; **p < 0.01; ***p < 0.001). *p*-values are calculated using either two-tailed unpaired Student’s *t*-test or ANOVA followed by *post-hoc* Tukey test.

### Data Availability

All described data are contained in the manuscript.

## Results

### Oxytocin Enhances Phospho-TrkB in Cortical Neurons

**Figure 1:**
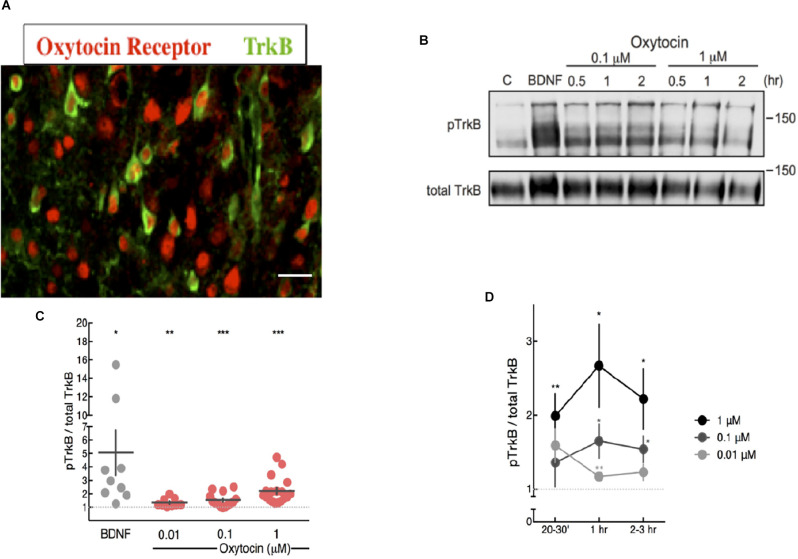
Oxytocin ligand increased pTrkB level in primary cortical neurons detected by immunoblotting. **(A)** Expression of oxytocin and TrkB receptors in cortical neurons of 2 week mice. Each receptor was probed with specific antibodies for oxytocin receptor, red (Mitre et al., 2016) and TrkB, green (Bath et al., 2008). The BD transduction 610102 antibody was used to detect TrkB. Potential staining of OXTR in the nucleus of some cells can be visualized, which has been previously reported (Kinsey et al., 2007). **(B)** Oxytocin induces activation of TrkB receptors in primary cortical neurons, assessed by western blot with anti-pTrkB (Chao lab) and TrkB antibodies (R & D AF1494) BDNF is a positive control. **(C,D)** Time course and dose-response plot of quantitative analysis of densitometric immunoblot scans from 11 independent experiments, with oxytocin treatments of 0.01, 0.1 and 1 mM for 20–30 min. The pTrkB/total TrkB ratio was calculated by taking the ratio of the pTrkB densitometric signal over the total TrkB densitometric signal for each sample and then represented as a value relative to untreated control (BDNF: 6.95 ± 2.41 fold above control, *N* = 9, *p < 0.03; oxytocin 0.01 μM: 1.34 ± 0.10, *N* = 9, **p < 0.006; oxytocin 0.1 μM: 1.53 ± 0.13, *N* = 16, ***p < 0.001; oxytocin 1 μM: 2.22 ± 0.23, *N* = 18, ***p < 0.0001; oxytocin 10 μM: 1.21 ± 0.03, *N* = 4, ***p < 0.0006). The *p* value (treatment vs. control) was determined with ANOVA test followed by *post-hoc* Tukey (*p < 0.05, **p < 0.01). **(D)** Time course and dose-response plot of quantitative analyses of densitometric immunoblot scans from 11 independent experiments withoxytocin treatments of 0.01 μM, 0.1 μM, and 1 μM lasting either 20–30 min, 1 h, or 2–3 h.

Neurotrophins, such as NGF and BDNF, activate Trk receptor tyrosine kinases through autophosphorylation and intracellular signaling. Activation of Trk receptor tyrosine kinases can also occur *via* a GPCR mechanism, without the involvement of neurotrophins (Lee and Chao, 2001; Lee et al., 2002a, b). We tested the possibility that oxytocin, a neuropeptide from the hypothalamus might activate Trk tyrosine kinase ability. Since TrkB is a predominant neurotrophin receptor expressed in the central nervous system, we assayed cortical neurons in culture. Detection of TrkB and oxytocin receptors in the cortex were conducted using specific antibodies against the intracellular domains of TrkB and oxytocin receptors. Immunohistochemical analysis indicated cortical regions contained between 15% and 30% expression for the oxytocin receptor (Mitre et al., 2016). A similar distribution has been observed for the TrkB receptor (Bath et al., 2008). Co-expression of TrkB and oxytocin receptors was observed in sections taken at 2 weeks of age (Figure 1A).

Treatment of primary cortical neurons with oxytocin was followed by measurements of TrkB phosphorylation with phospho-antibodies at tyrosine site 816, which are specific for TrkB and not other receptor tyrosine kinases. The dose of oxytocin is based upon conditions used by McCarthy (1990), who administered oxytocin intracerebroventricularly in female mice to study maternal effects upon infanticide. Also, Ceanga et al. (2010) established conditions in hippocampal primary neurons to study the effects of oxytocin upon cultured neurons subjected to oxygen-glucose deprivation (OGD). In this study, a dose of oxytocin between 0.1 and 100 μM was tested.

Mouse primary cortical neurons were treated with oxytocin at different concentrations from 0.01 to 1 μM. TrkB phosphorylation was detected at all concentrations in varying degrees (Figure 1B). Phosphorylation of TrkB *via* oxytocin was observed from 30 min to 2 h and required at least 1 h at the lowest concentration of oxytocin (0.01 μM). For these studies, we used a pTrkB antibody that recognized a C-terminal phosphotyrosine site at Y816 (Arevalo et al., 2006). This antibody did not cross-react with non-phosphorylated or phosphorylated TrkA NGF receptor and was extensively characterized in mouse tissues for specificity and efficacy (Bath et al., 2008).

Curiously, the dose effects of oxytocin upon TrkB displayed an inverted-U shape in terms of concentration and time course, with higher concentrations and longer treatments leading to a lower level of transactivation of TrkB (Figures 1C,D). These results are consistent with previous reports of a biphasic profile in studies of the dose of oxytocin in social behavior. Studies on the consequences of social reward indicated oxytocin stimulated social preference at low concentrations but became lost at higher concentrations (Borland et al., 2019). The *U*-shaped curve usually results from a nonlinear relationship between ligand and receptor. Given we report a biphasic curve in our studies of oxytocin and pTrkB transactivation, this relationship may be relevant to the mechanism of oxytocin receptor signaling and the oxytocin concentrations and what is necessary for intranasal application. The U-shape model has been invoked to explain the relationship between brain oxytocin levels and neural activity in humans. Depending upon the dose of oxytocin, differences in social reward can occur and mediate social interactions.

### Mouse Brain Slices Treated With Oxytocin

To investigate the generality of oxytocin’s effects beyond primary neuronal cultures, we obtained acute brain slices from animals in the second week of postnatal life when the highest oxytocin and TrkB receptor expression were observed. Also, receptors for oxytocin and BDNF overlap in their distribution in brain regions and are found in the same cortical layers in the cortex during this period of development (Cellerino et al., 1996; Mitre et al., 2016).

**Figure 2:**
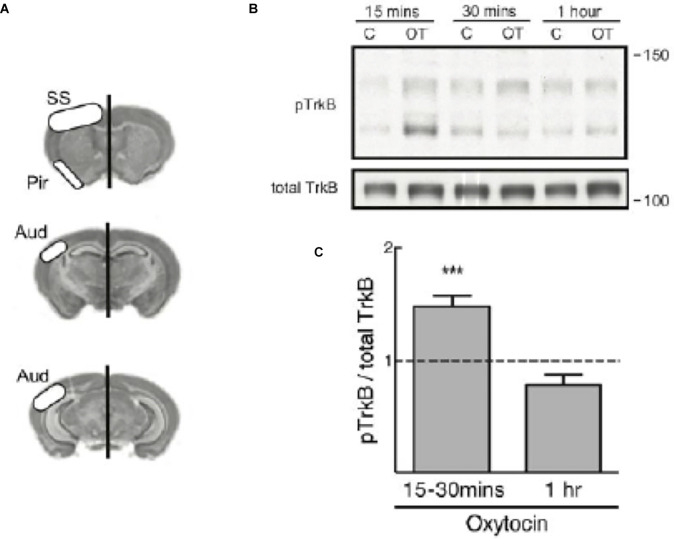
TrkBphosphorylation is elevated in mouse cortical slices treated withoxytocin. **(A)** Diagram regions of the slice dissection.**(B)** A representative immunoblot for TrkB phosphorylation(pTrkB from Chao lab) from brain slices treated with oxytocin (OT) at 1 μM for different time course (upper band is size of full length TrkB). Total TrkB, R & D AF1494. **(C)** The graph demonstrates pTrkB levels in mouse cortical tissue slices (*n* = 12). Summary plot of quantitative analyses of densitometric immunoblot scans from 12 independent experiments with oxytocin treatments at 15–30 min or 1 h. pTrkB/total TrkB ratio is reported and was calculated by taking the ratio of the pTrkB densitometric signal over the total TrkB densitometric signal for each sample, and then represented as a value relative to untreated control (oxytocin 15–30 min: 1.39 ± 0.08, *N* = 12, ***p < 0.0001; oxytocin 1 h: 0.69 ± 0.04, *N* = 3, *p* > 0.06). The *p* value was determined with ANOVA test followed by *post-hoc* Tukey (***p < 0.01).

Therefore, we treated 350 μm thick acute brain slices with 1 μM oxytocin at different timepoints and assessed the transactivation of TrkB. Each hemisection slice was placed in a separate chamber, one serving as the untreated control chamber and the other as the oxytocin treatment chamber. Slices were incubated in oxygenated artificial cerebrospinal fluid (ACSF) and collected at 15 min, 30 min, or 1 h after oxytocin treatment or incubation alone in the control chamber. Tissue from each condition was collected from the somatosensory cortical area, piriform cortex, and auditory cortex. Lysates were prepared and processed for immunoblotting to probe for the levels of phospho-TrkB (Y816) and total TrkB, as shown in the schematic in Figure 2A.

A time course from 15 min to 1 h showed a modest increase in pTrkB, in its full length form at approximately 140 kDa (Figure 2B). A quantitative analysis of densitometric scans of immunoblots from twelve independent experiments with oxytocin was undertaken (Figure 2C). We quantitated the ratio of pTrkB to total TrkB and found the value was elevated, but declined with time. Staining of the cortical sections demonstrated oxytocin receptors were abundantly expressed (Figure 2A).

In this analysis, two forms of TrkB were visualized by immunoblotting. Interestingly in this preparation, a smaller TrkB precursor at 110 kDa was observed which was detected after oxytocin treatment. The smaller isoform represents an unprocessed and underglycosylated species. Indeed, the smaller TrkB isoform protein was detected with the phospho-TrkB Y816 antibody and has been associated with TrkB receptors found in intracellular locations. The smaller unprocessed form of TrkB has been observed in other studies (Watson et al., 1999) and is different from a truncated form of TrkB (called TrkB.T1), which lacks the cytoplasmic and catalytic domain (Carim-Todd et al., 2009). The pTrkB (Y816) antibody does not recognize the truncated TrkB.T1 isoform, which lacks the Y816 site.

It has been noted that post-translational glycosylation of Trk receptors is required for localization at the cell surface. The precursor form of TrkB lacking glycosylation is phosphorylated intracellularly (Watson et al., 1999), indicating TrkB receptors can undergo activation both in internal membranes and the cell surface. From previous studies, the processing and maturation of the TrkB receptor represent crucial elements in receptor localization and the transactivation process (Watson et al., 1999; Schecterson et al., 2010). Transactivation of TrkB is observed in primary cultures and tissues from cortical brain slices of 2-week old mice treated with oxytocin (Figure 2B), indicating that this process occurs *in vivo* at the developmental peaks of oxytocin receptor expression.

### TrkB Transactivation Occurs in the Absence of BDNF

One question with transactivation receptors is whether endogenous neurotrophin ligands themselves contribute to receptor phosphorylation. The appearance of intracellular TrkB receptors by oxytocin suggests that transactivation might involve the BDNF ligand. For example, oxytocin might increase the synthesis and/or release of BDNF, which could account for the effects of TrkB phosphorylation. It is well known that BDNF is produced by many neurons in an activity-dependent manner.

**Figure 3:**
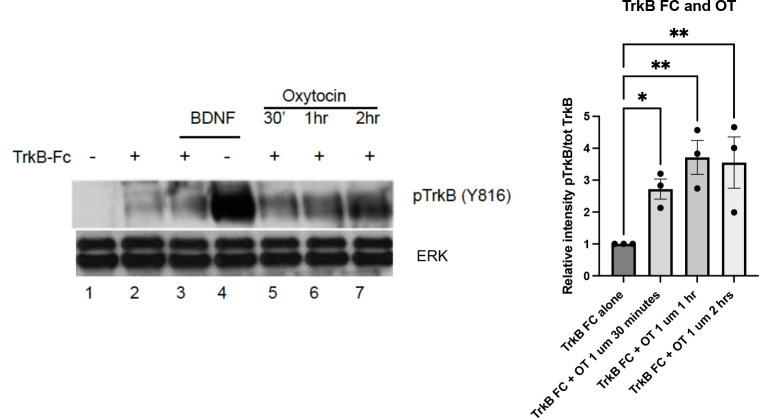
TrkB transactivation by oxytocin is independent of the presence of BDNF. Cortical neurons were treated with 0.1 μM oxytocin (OT) for 0.5, 1, and 2 h in the presence of TrkB-FC (100 ng/ml). Three independently treated sets of neurons were assessed for responsiveness. BDNF (50 ng/ml) is used as positive control in this experiment. Lane 1, no treatment; Lane 2, TrkB-Fc; Lane 3, BDNF and TrkB, Fc; Lane 4, BDNF alone; Lanes 5-7, TrkB-Fc at 30’, 1 h, 2 h. Lysates were prepared from primary cultures of cortical neurons and run *via* Western blotting to probe for pTrkB Y816 (from Chao lab) and total ERK levels (Cell Signaling #9102). The graph (right) illustrates the relative density of pTrkB which was calculated by the ratio of the pTrkB densitometric signal over the total ERK densitometric signal for each sample, and then represented as a value relative to untreated control. The significance of TrkB-Fc + oxytocin treatment from the three time points was calculated with one way ANOVA pairwise comparison.

To determine whether BDNF is present during the oxytocin-induced TrkB phosphorylation, we utilized a TrkB-Fc chimeric protein that is effective in binding and effectively scavenging BDNF. The TrkB-Fc chimeric protein has been utilized to sequester BDNF in primary cortical cultures in microarray experiments. In the absence of BDNF, this treatment identified genes which were affected by a loss of BDNF (Mariga et al., 2015). In this experiment, we first confirmed that the application of the TrkB-Fc protein was capable of lowering BDNF levels and preventing TrkB phosphorylation (Figure 3, lanes 2–3). A robust induction of TrkB phosphorylation through its ligand, BDNF (lane 4) was greatly decreased if cells were concomitantly treated with the TrkB-Fc fusion protein (lane 3), demonstrating the efficacy of TrkB-Fc in scavenging BDNF (50 ng/ml) in the media. In contrast, treatment with TrkB-Fc did not prevent oxytocin from elevating phospho-TrkB levels in primary cortical neurons (Figure 3, lanes 5–7).

In the presence of TrkB-Fc, oxytocin was still capable of eliciting an increase in phospho-TrkB. The increase in pTrkB in this treatment was lower than the effect of BDNF alone (lane 4), which is consistent with other experiments. A quantitation of the TrkB-Fc treatment compared with the effects of TrkB-Fc plus oxytocin is shown in Figure 3. The analysis supports oxytocin’s ability to activate TrkB receptors independent of the presence of BDNF. Together these results demonstrate that oxytocin can transactivate TrkB receptors in the absence of BDNF.

### Heterologous Expression of Oxytocin and TrkB Receptors Recapitulates Transactivation

**Figure 4:**
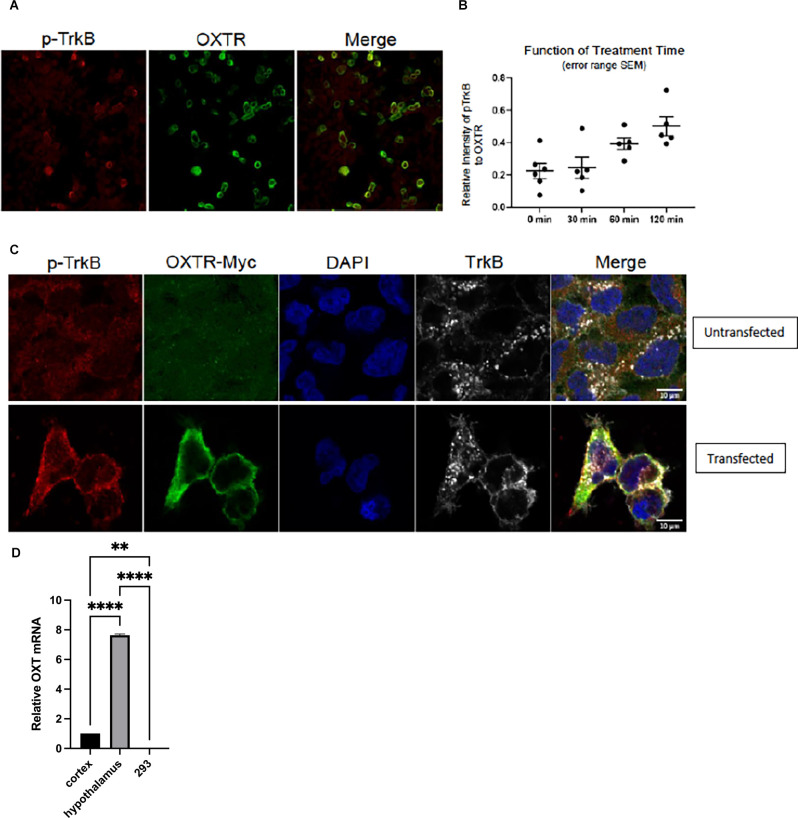
Transfection of OXTR plasmid in HEK-293-TrkB cells produces increased phospho-TrkB. Immunocytochemistry of cells with pTrkB in red transfected with myc-tagged OXTR in green. Co-expression of OXTR led to activation of TrkB. Next day, cells were serum starved for 2 h and then treated with 1 μM oxytocin. Images were taken at 120 min of oxytocin treatment. **(A)** Left panel, staining with phosphor-TrkB Y816 Cell Signaling 4168 antibody (red). Middle panel, staining with myc-tagged OXTR (green; 18). Right panel, Merge of pTrkB and OXTR. **(B)** Images from time points of 0 (no oxytocin), 30, 60 and 120 min were collected with matching laser intensities and detector responses. The results were quantitated by Image J analysis as a ratio of Red (pTrkB) to Green (OXTR) staining. The results were plotted in GraphPad Prism 8.2.0. Zeiss CZI files were imported into Image J using Bio-formats 6.5.0. **(C)** Upper panel, untransfected HEK293/TrkB cells. Staining with antibodies against p-TrkB (Cell Signaling 4168); OXTR-myc, DAPI and total TrkB (R & D System AF1494) is shown.Lower panel, HEK293/TrkB cells are transfected with 0.5 μg/well myc-taggedOXT receptor plasmid for 16 h. The effect of OXTR expression on TrkB activation in cells was evaluated by detecting the levels of p-TrkB (Y816), leftlower panel and Merge.**(D)** Negligible oxytocin ligand RNA is detected in HEK293-TrkB cells.Expression of oxytocin ligand RNA is measured by RT-qPCR from total RNAisolated from cortex, hypothalamus, and HEK293-TrkB cells. Relative expressionlevels of OXT mRNAs was normalized to actin, statistical calculations were madeusing one-way ANOVA with multiple comparisons (Prism 9).

To extend the breadth of these results, we turned to an *in vitro* assay to evaluate whether oxytocin receptor transactivates TrkB. This was done to test whether the transactivation event could be observed in a heterologous background. This is an important question, as it addresses whether these receptor molecules are sufficient for transactivation. Therefore, the HEK293 human cell stably expressing TrkB (Narisawa-Saito et al., 2002) was employed to investigate the link between oxytocin and TrkB receptors in a heterologous non-neuronal cell background. We found that transfection of an expression plasmid for oxytocin receptor in HEK293-TrkB cells gave rise to an increase in phospho-TrkB by immunohistochemical analysis (Figure 4A), similar to the time course of primary cortical neurons treated with oxytocin shown in Figure 1. A quantitation of the time course indicated the phospho-TrkB appeared after 30 min (Figure 4B). This interval of time is slower than the activation by BDNF. The time course is consistent with other transactivation events by GPCR receptors (Lee and Chao, 2001).

In order to verify the role of oxytocin receptors during transactivation, we carried out an immunofluorescence analysis of oxytocin and TrkB receptors. Introduction of a myc-tagged oxytocin receptor vector in HEK293-TrkB cells gave rise to strong expression, which was effectively visualized with a myc-antibody (Figures 4A,C). In this case, the transfected oxytocin receptor was expressed in the heterologous HEK293 non-neuronal kidney cell line. The oxytocin ligand was not present in several conditions where phospho-TrkB was activated, suggesting the oxytocin GPCR receptor possesses an activity in the absence of the oxytocin ligand (Figure 4D).

The transfected cells were tested for the state of phospho-TrkB (Y816) receptors. In these *in vitro* experiments, only low levels of endogenous expression of phospho-TrkB were observed in HEK293-TrkB cells (Figure 4A). However, after transfection of the oxytocin receptor, an increase in cells co-expressing both oxytocin and pTrkB receptors occurred, as visualized by increased fluorescence of oxytocin receptor and phospho-TrkB in membrane and cytoplasmic localizations (Figure 4C). DAPI staining was used to follow the localization of the receptors in these cells. At higher magnification, positive cells were visualized by merging the staining with antibodies against the phospho-TrkB and oxytocin receptors. Abundant staining of both receptors was observed in cells monitored in Figure 4C, indicating the appearance of transfected oxytocin receptors was associated with the activated phospho-TrkB receptors.

The results of the HEK293-TrkB experiments recapitulated the activation of phosphorylated TrkB receptors observed in primary neurons presented in Figure 2. In these experiments, the expression of oxytocin receptors had a striking impact without exogenous application of oxytocin ligand. It is formally possible that low levels of oxytocin may exist in serum used for cell culture, as has been frequently documented.

One explanation for the apparent ligand independence of transactivation is that expression of endogenous oxytocin ligand might occur in heterologous cells. To address this possibility, we carried out measurements of oxytocin ligand RNA isolated from cortex and hypothalamus and HEK-293TrkB cells by RT-qPCR. Primers for the human transcript were used to assay HEK-293 cells and primers for mouse oxytocin mRNA were used for the mouse cortex and hypothalamus.

Negligible levels of oxytocin message were found in the HEK-293 cells by RT-qPCR measurements (Figure 4D). In contrast, oxytocin ligand RNA was found to be prevalent in the mouse hippocampal tissues.

In order to elucidate the role of oxytocin receptors during transactivation, we carried out an immunofluorescence analysis of oxytocin and TrkB receptors. The introduction of a myc-tagged oxytocin receptor vector in HEK293-TrkB cells gave rise to ample expression, which was effectively visualized with a myc-antibody (Figures 4A,C).

The transfected cells were tested for phospho-TrkB (Y816) receptors. In these *in vitro* experiments, only low levels of endogenous expression of pTrkB were observed in HEK293-TrkB cells (Figure 4A). However, after transfection of the oxytocin receptor, an increase in cells co-expressing both oxytocin and pTrkB receptors occurred, as visualized by increased fluorescence of oxytocin receptor and pTrkB in membrane and cytoplasmic localizations (Figure 4B). DAPI staining was used to follow the localization of the receptors in these cells. At higher magnification, positive cells were visualized by merging the staining with antibodies against the phosphor-TrkB and oxytocin receptors. Abundant staining of both receptors was observed in cells monitored in Figure 4C, indicating the appearance of transfected oxytocin receptors was associated with the activation of pTrkB receptors. In particular, the intracellular localization of pTrkB was seen in Figure 4C, as was reported by other forms of activation (Rajagopal et al., 2004; Schecterson et al., 2010).

### Specificity of Transactivation

To assess the specificity of the oxytocin receptor’s transactivation activity toward TrkB, we assessed the ability of a related vasopressin receptor in this assay. Vasopressin has three receptor isoforms, V1aR, V1bR, and V2R, which belong to the family of seven transmembrane receptors with highly similar sequence identity to the oxytocin receptor (Thibonnier et al., 1994). About 25% of the sequences are the same between the receptors for vasopressin (V2, V1a, V1b) and oxytocin. We originally chose four peptides in the oxytocin receptor sequence which diverged from the vasopressin receptor V1a and V1b, described previously (Marlin et al., 2015; Mitre et al., 2016). The regions were located in the N- and C-termini and the intracellular loops, which displayed very low homology with the vasopressin receptor sequences.

**Figure 5:**
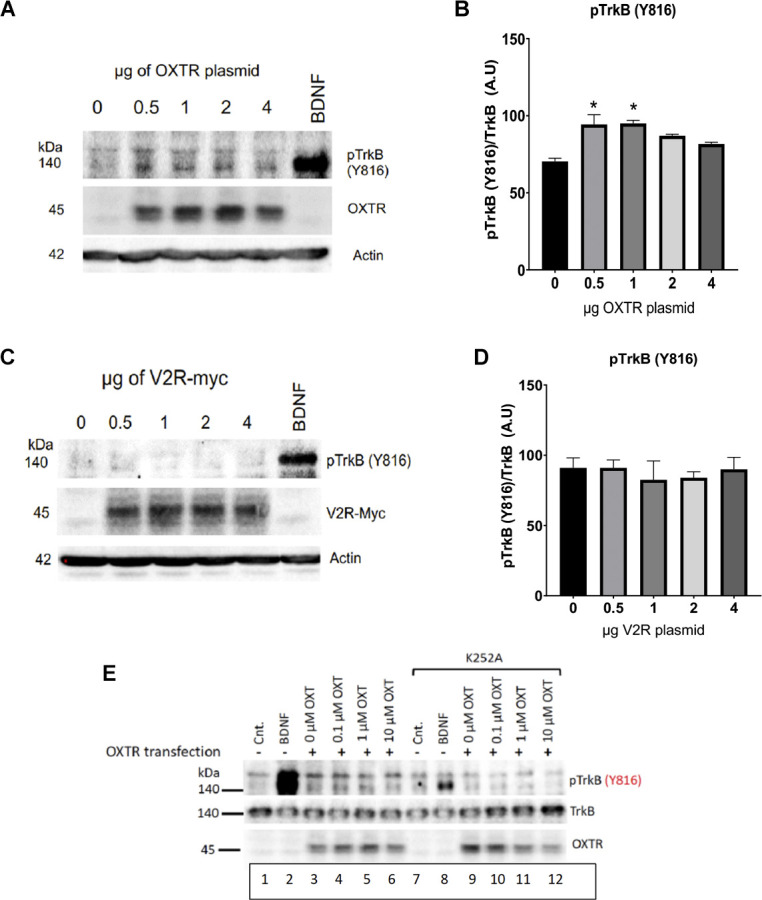
Oxytocin receptor (OXTR), but not vasopressin receptors (V2R), induces TrkB phosphorylation. HEK-293-TrkB cells were transfected with increasing amounts of OXTR **(A)** and V2R **(B)** plasmids for 16 h and then the levels of pTrkB-Y816 (Cell Signaling 4168) were detected by Western blot. BDNF treatment was used as a control. Quantitation of pTrkB from increasing plasmid amounts is shown plots **(C,D)**. All *p* values were determined with ANOVA test followed by *post-hoc* Tukey (*p < 0.05). **(E)** Treatment with K252a. The Trk inhibitor, K252a (Berg et al., 1992) at a concentration of 200 nM blocks BDNF and oxytocin-mediated transactivation of TrkB receptors. Lanes 2 and 8, BDNF treatment. Lanes 3-6 and 9-12, Transfection of OXTR. Lanes 7-12, Treatment with K252a. HEK293-TrkB cells are transfected with oxytocin receptor (OXTR) and monitored for expression by western blot. Controls (Cnt) are untreated cells, lanes 1 and 7. OXT, oxytocin ligand.

Transfection of a myc-tagged V2R construct gave a sizeable level of vasopressin receptor expression in heterologous HEK293-TrkB cells, however, the appearance of activated p-TrkB was not observed using anti-Y816 TrkB antibodies (Figure 5B). Transactivation of TrkB occurred in presence of the oxytocin receptor (Figure 5A), but not the related V2R vasopressin receptor (Figure 5C). The lower protein of 140 kDa in Figure 5A corresponds to TrkB, which also appeared after BDNF treatment. Quantitation of responses of TrkB receptors is shown in Figures 5B,D which validates a preferential effect of oxytocin. Transactivation of TrkB occurred preferentially through the oxytocin receptor, in contrast to the closely related vasopressin V2R receptor.

To establish further that TrkB is a target of transactivation, we resorted to the K252 inhibitor of Trk receptors. Figure 5E shows that blocking pTrkB results in a diminution of transactivation by oxytocin. Transfection of OXTR was required; BDNF was used as a control. The HEK293-TrkB cell system recapitulated the activation of phosphorylated TrkB receptors observed in primary neurons presented in Figure 2. The results also indicate the oxytocin and BDNF TrkB receptor proteins are sufficient to observe transactivation in heterologous HEK293 cells.

### Effects of Oxytocin on Gephyrin

Common downstream targets of BDNF and oxytocin have been observed in a comprehensive siRNA screen (Wuchter et al., 2012) and bioinformatics (Chatterjee et al., 2016). Among the identified proteins were TrkB, TrkC, gephyrin, and members of the MAPK and mTOR pathways. To focus on functional proteins relevant to both ligands, we chose proteins involved in inhibitory pathways in the nervous system. It has been established that oxytocin acts to reduce inhibition in many brain areas, such as the auditory and piriform cortex and the hippocampus (Owen et al., 2013; Mitre et al., 2016). Maturation of inhibitory neurons also occurs through BDNF signaling *via* TrkB receptors which in turn promotes the assembly of GABAergic synapses and their maintenance (Rico et al., 2002; Park and Poo, 2013). How do oxytocin and BDNF coordinate their responses? Receptors for oxytocin and BDNF are expressed in the same subpopulation of cells in the cortex at a time of developmental inhibitory circuit maturation that is mediated by BDNF. These observations led us to test the hypothesis that oxytocin may interact with TrkB signaling to modulate inhibitory circuits.

To assess the impact of oxytocin on inhibitory proteins, we measured the change in fluorescence and accumulation of gephyrin, a protein that helps to aggregate GABA ion channels. As a scaffold protein, gephyrin affects GABA_A_R clustering and results in the loss of inhibitory postsynaptic currents (Kasaragod and Schindelin, 2018). Primary cultures of cortical neurons were therefore established to examine the distribution of gephyrin using immunofluorescence. We identified interneurons by staining them with parvalbumin and somatostatin. These cells were co-stained with oxytocin receptors at a frequency of 35%–40%.

**Figure 6:**
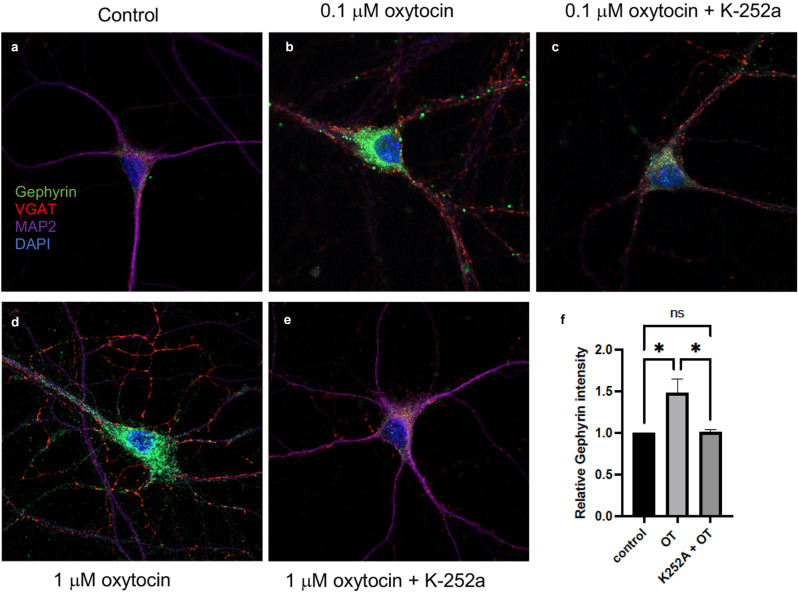
Oxytocin increased gephyrin clustering in primary cortical neurons (DIV7). Immunostaining of Gephyrin (green; Abcam ab25784), VGAT (red; Synaptic Systems 131011), MAP2 (Covance PCK554050), DAPI (blue) immunoreactivity was detected in primary cortical neurons. **(A)** Image of staining of control cells. No treatment. Scale, 10 μm. **(B)** Image of 0.1 μM oxytocin-treated cells for 2 h. Scale, 10 μm. **(C)** 0.1 μM oxytocin plus K252a (200 ng/ml). **(D)** 1 μM oxytocin (2 h). Scale, 10 μm. **(E)** 1 μM oxytocin pretreated with 200 ng/ml K252a (2 h). **(F)** Summary plot of gephyrin fluorescence over the same size region of the cell body in response to oxytocin (OT). Three independent experiments were carried out with analysis of at least 50 cells across conditions for each experiment. The images were collected with matching laser intensities and detector response and subjected to ANOVA. All *p* values were determined by one-way ANOVA with multiple comparisons, *p* < 0.01.

After oxytocin stimulation for 2 h, a significant increase in the fluorescence of gephyrin was observed (Figure 6), which was normalized to each cell’s soma size. Two concentrations of oxytocin (0.1 and 1 μM) were applied to primary cultures, consistent with earlier experiments (Figure 6). The intensity of gephyrin fluorescence was clearly enhanced in the cell bodies after oxytocin treatment.

To determine whether the increased fluorescence of gephyrin was regulated upon Trk receptor signaling, we applied K-252a, the inhibitor of Trk receptors (Berg et al., 1992). After pre-incubation of cortical neurons with K-252a for 1 h, either 0.1 or 1 μM oxytocin was added to the neurons. Treatment with K-252a resulted in a decrease in the fluorescence of gephyrin by oxytocin at both concentrations, which was evident by immunostaining (Figures 6C,E). Quantitation of immunofluorescence over the cell body area was carried out (Figure 6F). The effect of oxytocin was compared in the presence and absence of K-252A. A marked effect of K-252a was observed upon gephyrin fluorescence.

To verify the inhibitory activity of K-252a, lysates from HEK293-TrkB cells were analyzed. Cells were treated with BDNF, in the presence and absence of K-252a (200 nM). A significant reduction of pTrkB induced by BDNF was observed with K-252a treatment. As another test, increasing concentrations of oxytocin were applied to elicit transactivation. Application of the K-252a inhibitor resulted in a decrease in the level of transactivation of pTrkB (Y816) receptors in cells treated separately with oxytocin (Figure 6).

Inhibition of TrkB resulted in a decrease of gephyrin expression by oxytocin. One explanation for the effects of oxytocin and TrkB upon the gephyrin protein is that oxytocin acts by transactivation. This possibility is supported by previous findings that BDNF is capable of promoting gephyrin expression and clustering (Mou et al., 2013; Gonzalez, 2014). The localization of gephyrin is critical to producing changes of GABAergic transmission. The experiment here indicated that oxytocin can alter gephyrin levels and its localization. At the same time, BDNF also acts upon the aggregation of gephyrin (Wuchter et al., 2012). The predominant effect of oxytocin modulation is to reduce inhibitory transmission, which is regulated by TrkB signaling. Because gephyrin is a target of both oxytocin and BDNF, we speculate that disinhibition in the central nervous system can occur through gephyrin modulatory signals from oxytocin and BDNF crosstalk. This event may serve as an important control point for interneurons to regulate inhibitory synaptogenesis.

## Discussion

Here we report a striking link between oxytocin action and BDNF signaling through its TrkB receptor, suggesting oxytocin may utilize downstream TrkB signaling for its actions. Other studies have maintained a regulatory role of BDNF in the expression of oxytocin that results in specific behavioral changes (Maynard et al., 2018). Oxytocin signaling plays a role in the pathophysiology of depression, schizophrenia, anxiety, and cognition, and interestingly, these same conditions are found to display lower levels of BDNF (Autry and Monteggia, 2012). It is conceivable that the actions of oxytocin and BDNF overlap biochemically and genetically Further studies of the cell biological and physiological consequences of oxytocin transactivation will help shed more light to the underlying receptor mechanisms of this far-reaching neuropeptide in the brain. The link of BDNF with oxytocin expands the possible mechanisms which are relevant to autism spectrum disorder, as well as other neuropsychiatric disorders.

In this study, we demonstrate oxytocin activates the TrkB receptor tyrosine kinase in brain slices, primary cortical neurons, and heterologous cells. Oxytocin did not induce new production of BDNF. Low levels of activated p-TrkB were also seen in presence of oxytocin receptors but in the absence of added oxytocin ligand. Oxytocin signal transduction events are not fully defined in terms of specific G proteins or β-arrestin involvement, which also participate in reproductive tissues and decision making and electrophysiological events in the brain. There are currently clinical trials of oxytocin in autism spectrum disorder, which have given uncertain outcomes. Our findings indicate actions of oxytocin receptors in the brain are likely to involve crosstalk with other signaling pathways. Additionally, transactivated TrkB receptors reside intracellularly, in contrast to the cell surface receptors induced by BDNF. Because of BDNF’s central role in the regulation of synapses, plasticity, physiology, and higher cognitive functions, transactivation of TrkB receptors by oxytocin represents an important developmental mechanism for organizing complex downstream signaling cascades.

There are several mechanisms that may account for TrkB transactivation. Calcium influx through voltage-sensitive calcium channels contributes to receptor tyrosine kinase transactivation. In this regard, removing Ca^2+^ blocks transactivation. Also Src family tyrosine kinases, such as the Fyn kinase, are sufficient to allow transactivation of Trk (Lee et al., 2002a, b). Treatment by the Src PP1 inhibitor decreases transactivation. Interestingly inhibition of Trk transactivation occurs after cycloheximide or actinomycin treatment implicating active biosynthetic processes requiring ongoing RNA and protein synthesis. As transactivation of Trk receptors is frequently found in intracellular localizations, this observation goes against the notion that internalization of cell surface receptors halts intracellular signaling (Rajagopal et al., 2004; Sorkin, 2005).

Several receptor tyrosine kinases, such as the EGF receptor, are targets for transactivation through oxytocin (Lin et al., 2012). The rationale for centering our attention on the BDNF TrkB receptor stems from its important role as a trophic factor responsible for neuronal survival, memory formation, and synaptic plasticity. Increases in BDNF are associated with cognitive improvement and alleviation of depression and anxiety. As a survival factor, BDNF supports the health of neurons and possesses key peripheral and sensory functions during development. Secreted in many tissues and cell types, including endothelial cells, astrocytes, lymphocytes, platelets, megakaryocytes, and skeletal muscle, BDNF is highly sensitive to activity-dependent changes (Park and Poo, 2013) and mediates many decisions that impact growth, synaptic consolidation, and higher cognitive functions.

In addition to TrkB receptors, The *Bdnf* gene is also highly regulated by at least nine distinct promoters that initiate transcription of multiple BDNF mRNA transcripts. Each transcript contains a 3’ coding exon that contains the open reading frame for the BDNF protein. In a noteworthy study, ablation of the first two promoters in the BDNF gene resulted in an impairment of maternal care, giving rise to a failure of pup retrieval (Maynard et al., 2018). This response is very similar to studies of oxytocin, where pup retrieval is closely associated with oxytocin levels (Marlin et al., 2015).

In the study by Maynard et al. (2018), a conditional deletion of TrkB receptors in oxytocin-expressing neurons was generated. This alteration led to changes in gene expression by oxytocin, resulting in impaired maternal care. In line with these findings, an increase in oxytocin levels was found to produce an increase in pup retrieval (Marlin et al., 2015). The TrkB BDNF receptor is highly expressed in oxytocin neurons and is responsible for synaptic transmission and plasticity. Based on these studies, disturbances in BDNF signaling have a profound impact on gene expression pathways responsible for plasticity in oxytocin neurons.

An interesting cell biological aspect is that the activation of receptors may occur independently of ligand (Cornejo et al., 2021). The localization of GPCR and Trk receptors in intracellular membranes may reflect excessive expression, dimerization, heterodimerization, or oligomerization of the receptors contribute to receptor function (Ribeiro et al., 2021). Oligomerization and subsequent activation of cell surface receptors may be affected by the cytoskeleton or lipid constituents in the membrane. Thus receptor activation is not an isolated molecular event mediated solely from a ligand-receptor interaction, but instead involves orchestrated interactions between the receptors and other cellular components. In the case of transactivation, allosteric changes in the receptor protein or its stoichiometry may promote the formation of receptor complexes and conformational changes that stimulate receptor signaling without ligand.

What are the consequences of TrkB transactivation by oxytocin? While both oxytocin and BDNF have individual mechanisms of action, transactivation allows oxytocin to employ novel signaling pathways downstream of BDNF TrkB receptors. Activity-dependent synaptogenesis in inhibitory neurons is driven by BDNF signaling *via* TrkB receptors (Rico et al., 2002; Kasaragod and Schindelin, 2018). At the same time, oxytocin changes plasticity by decreasing inhibition in central neurons (Wuchter et al., 2012; Marlin et al., 2015). An explanation for the impact of oxytocin and BDNF may come from inhibitory scaffold proteins, such as gephyrin, which can be activated by BDNF or oxytocin.

Gephyrin is a nodal point for aggregating GABA receptors, which mediate fast inhibitory neurotransmission in the brain. BDNF signaling through TrkB promotes an association of GABAA receptors with gephyrin and regulates gephyrin protein levels over time (Mou et al., 2013; Gonzalez, 2014). GABAergic synapses may be promoted by gephyrin clustering. Indeed, given the multidisciplinary action of these ligands, there is a strong connection between BDNF signaling, gephyrin clustering, and GABA signaling.

During neurodevelopment, excitatory responses are balanced by physiological changes in inhibition (Owen et al., 2013; Marlin et al., 2015). Neuromodulation may be explained by a balance of excitation and inhibition and by trophic factors and posttranslational scaffolding events in inhibitory neurons. What remains to be determined is how the dis-inhibitory effects are triggered by oxytocin binding to inhibitory cells. This is a pertinent question as previous findings in hippocampal slices revealed there is oxytocinergic depolarization of hippocampal interneurons (Owen et al., 2013). Neuromodulation may be explained by a balance of excitation and inhibition and by trophic factors and posttranslational scaffolding events in inhibitory neurons. In this case, we hypothesize that oxytocin might take advantage of TrkB signaling for the modulation of GABAergic neurons and for the regulation of axonal dynamics and synapse formation.

In addition to developmental events, BDNF has a role in synaptic plasticity, obesity, addiction, and many neuropsychiatric disorders. The large number of interrelationships with these disorders make it difficult to assign a mechanism to each case. However, the spatial location and the mode of timing, viz-a-vie acute vs. chronic application of BDNF, can give widely different results (Ji et al., 2010). Depending upon the dose and the method of application, a completely different time course response by BDNF on changes in spine formation, ERK, TrkB, LTP activities, and immediate early gene expression (Ji et al., 2010). This suggests that the activation of TrkB by oxytocin may give alternative outcomes based upon transient vs. sustained stimulation. Hence the concentration of oxytocin will likely provide different signaling kinetics by acute and gradual modes of exposure.

Since BDNF is a negatively charged protein, it does not pass through the blood-brain barrier, making therapeutic strategies a challenge for the treatment of neurological diseases. For this reason, transactivation of TrkB receptors represents an alternative method of triggering a trophic response. The strategies for applying neurotrophic factors in human neurological diseases are based on the potential of a beneficial recovery of injured or deprived neurons. This treatment implies not only cell survival, but also restoration of proper synaptic functioning of vulnerable neurons.

As GPCR ligands, adenosine and Pituitary adenylate cyclase-activating polypeptide (PACAP) can transactivate Trk receptors with a similar time course and mechanism (Lee et al., 2002a, b; Rajagopal et al., 2004; Sorkin, 2005). Transactivation elicited by GPCR ligands can produce neuroprotective effects, such as increased survival of PACAP-treated basal forebrain cholinergic neurons after axotomy (Takei et al., 2000), providing a method for increasing neurotrophin signaling in neurodegenerative diseases. Other ligands can transactivate Trk receptors, such as epidermal growth factor EGF (Puehinger et al., 2013), glucocorticoids (Jeanneteau et al., 2008), dopamine (Iwakura et al., 2008), and zinc (Huang et al., 2008). Unexpectedly, EGF signaling activates TrkB and TrkC in mouse embryonal precursor cells, providing a developmental mechanism for regulating their migration into the developing cortex (Puehinger et al., 2013). The interactions between glucocorticoid and BDNF signaling are important for eliciting neuroprotection (Jeanneteau et al., 2008), modulating target gene expression, and promoting neuronal plasticity in response to stress (Jeanneteau et al., 2012; Lambert et al., 2013).

Since small molecules are capable of targeting populations in the CNS, it is conceivable that oxytocin might use TrkBreceptor as a downstream signal to increase trophic support as well as dendritic and axonal branching and synaptogenesis. Hence in the future, oxytocin could potentially be considered as a treatment of neurological and psychiatric disorders through a transactivation mechanism with different G protein-coupled ligands.

## Data Availability Statement

The raw data supporting the conclusions of this article will be made available by the authors, without undue reservation.

## Author Contributions

MM, MC, and RF designed the research. MM, KS, KW, and LK performed the experimental research. MC and MM wrote the manuscript. All authors contributed to the article and approved the submitted version.

## Conflict of Interest

The authors declare that the research was conducted in the absence of any commercial or financial relationships that could be construed as a potential conflict of interest.

## Publisher’s Note

All claims expressed in this article are solely those of the authors and do not necessarily represent those of their affiliated organizations, or those of the publisher, the editors and the reviewers. Any product that may be evaluated in this article, or claim that may be made by its manufacturer, is not guaranteed or endorsed by the publisher.

## ABBREVIATIONS

OXTR: oxytocin receptor
GPCR: G protein-coupled receptor
BDNF: brain-derived neurotrophic factor
TrkB-Fc: fusion protein containing the extracellular domain of TrkB fused to Fc domain
V2R: vasopressin receptor
RT-qPCR: quantitative reverse transcriptionPCR
VGAT: vesicular GABA transporter
GABA: gamma-aminobutyric acid
K252a: alkaloid kinase inhibitor.
